# Predictive values of colorectal cancer alarm symptoms in the general population: a nationwide cohort study

**DOI:** 10.1038/s41416-019-0385-x

**Published:** 2019-02-22

**Authors:** Sanne Rasmussen, Peter Fentz Haastrup, Kirubakaran Balasubramaniam, Sandra Elnegaard, René dePont Christensen, Maria Munch Storsveen, Jens Søndergaard, Dorte Ejg Jarbøl

**Affiliations:** Research Unit of General Practice, Department of Public Health, University of Southern Denmark, J. B. Winsløws Vej 9A, 5000 Odense C, Denmark

**Keywords:** Colorectal cancer, Digestive signs and symptoms

## Abstract

**Background:**

Alarm symptoms are used in many cancer referral guidelines. The objectives were to determine the 1-year predictive values (PVs) of colorectal cancer (CRC) alarm symptoms in the general population and to describe the proportion of alarm symptoms reported prior to diagnosis.

**Methods:**

A nationwide prospective cohort of 69,060 individuals ≥40 years randomly selected from the Danish population was invited to complete a survey regarding symptoms and healthcare-seeking in 2012. Information on CRC diagnoses in a 12-month follow-up came from the Danish Cancer Registry. PVs and positive and negative likelihood ratios were calculated.

**Results:**

A total of 37,455 individuals participated (response rate 54.2%). Sixty-four individuals were diagnosed with CRC. The single symptom with the highest positive PVs (PPV) and LR+ was rectal bleeding. PPVs were generally higher among individuals aged ≥75 years and highest among those reporting at least one specific alarm symptom that led to a GP contact.

**Conclusion:**

In general, the PPVs of CRC alarm symptoms are low and the NPVs high, especially in the youngest age groups. The LR +  show a relative association with specific symptoms like rectal bleeding. Future campaigns on early diagnosis of CRC should focus on healthcare-seeking when experiencing rectal bleeding and target older people with the highest incidence.

## Background

Colorectal cancer (CRC) causes substantial morbidity and mortality throughout the world and the highest incidence is seen in developed countries with a Western culture.^1^ The prognosis of CRC is highly dependent on the stage of disease at diagnosis. The 5-year survival rate ranges from 90% for localised stage CRC to 10% for patients with distant metastases.^2^ To promote early diagnosis and improve survival rates, many countries have implemented cancer referral guidelines and fast track endoscopy for patients with alarm symptoms indicative of CRC.^3,4^ For CRC, alarm symptoms are specific symptoms such as rectal bleeding or changes in bowel patterns for individuals ≥40 years. However, non-specific symptoms such as weight loss and tiredness could be important markers of malignant disease and, in general, almost half of cancer patients present non-specific symptoms prior to diagnosis.^5^ In Denmark, screening for CRC is offered to people in the age of 50–74 years. However, only approximately 25% of all CRC are detected by screening.^6^ For the rest of the cases and in the group aged 75 years and older, symptoms will still have a significant role in the diagnostic process of CRC.

It has been demonstrated that both specific and non-specific symptoms of CRC are common in the general population and few consult their general practitioner (GP) when experiencing alarm symptoms of CRC.^7^ Positive predictive values (PPVs) for alarm symptoms of CRC have mostly been investigated for persons consulting their GP with alarm symptoms and little is known about the negative predictive values (NPVs).^8–10^ As campaigns to increase public awareness of alarm symptoms and promote earlier presentation by the patients are progressively carried out worldwide,^11^ it is important to gather knowledge about the PVs of alarm symptoms in the general population.

The aims of this study were (1) to determine the 1-year PV of specific and non-specific alarm symptoms of CRC in the general population 40 years or above and (2) to describe the proportion of specific and non-specific alarm symptoms reported by patients prior to diagnosis of CRC.

## Methods

### Study design and population

The study was designed as a nationwide cohort study based on questionnaires and national registries, imbedded in the Danish Symptom Cohort (DaSC).^12^ This particular study aimed to gather knowledge about the predictive values of specific and non-specific alarm symptoms of CRC in the general population. Other studies have already reported predictive values of gynaecological cancer alarm symptoms^13^ and upper gastrointestinal cancer.^14^ Parts of the methods described below have therefore been previously described.^7,12,15^

From the Danish Civil Registration System (CRS), 100,000 adults aged 20 years or above were randomly selected and invited to participate in a survey. All Danish citizens are registered in the CRS with a unique personal identification number. Prior to the sampling procedure, individuals were excluded if they had declared to the CRS that they did not want research-related inquiries. The individuals received a postal letter explaining the purpose of the study. The questionnaire was designed using the internet-based platform SurveyXact.^16^ A unique login for a secure webpage was included in the letter. This provided access to a comprehensive web-based questionnaire. To prevent exclusion of people with no access to the internet, participants were offered to complete the survey by telephone interview. When an invited subject was unable to respond due to severe illness or having moved abroad, family or relatives could decline the invitation on behalf of the invited person. The reason for not responding was then registered as illness or moved abroad.

### The questionnaire

The methodological framework for developing, pilot testing and field testing the questionnaire is described in detail elsewhere.^12^ This paper addresses the specific and non-specific symptoms indicative of CRC. These symptoms were selected based on a review of literature, national and international cancer referral guidelines and descriptions of cancer pathways.^3,4,17^ In total, ten predefined symptoms reported by individuals aged 40 years or above form the base of this paper (Table 1). The four specific symptoms and the age limit were chosen as it is included in the Danish cancer referral guidelines (Supplementary Figure 1). The respondents were asked whether or not they had experienced one or more of the symptoms in the preceding 4 weeks. The respondents were additionally asked whether or not they had contacted their GP regarding the symptom. The wording of the question regarding symptom experience was: “Have you experienced any of the following sensations, symptoms or discomfort within the past 4 weeks?” with the option to select one or more of the predefined symptoms. With regard to GP contact, the question was worded: “Have you contacted your general practitioner concerning the symptom(s) you have experienced within the preceding four weeks, by appointment, telephone or e-mail?” An item concerning when the symptom(s) occurred for the first time was also included. The response categories were: “less than one month ago”, “1–3 months ago”, “3–6 months ago” or “more than six months ago”.

**Table 1:** Specific and non-specific alarm symptoms of colorectal cancer

Specific alarm symptoms of colorectal cancer
Abdominal pain
Change in stool texture^a^
Change in stool frequency^a^
Blood in stool/rectal bleeding
Non-specific alarm symptoms of colorectal cancer
Diarrhoea
Constipation
Abdominal bloating
Weight loss
Feeling unwell
Tiredness

^a^Only experience of symptoms more than 1 month earlier was included

### Register data

Information on CRC diagnoses in the respondent cohort aged 40 years or above was retrieved from the Danish Cancer Registry (DCR). The DCR contains personal and tumour characteristics for all incident cancer cases in Denmark including date of diagnosis and ICD-10 codes for lesions.^18^ Only cases diagnosed in a 12-month period after the completion of the questionnaire were included. Furthermore, the cases were excluded if the individual had been diagnosed with the same ICD-10 code in a time period covering 5 years prior to the completion of the questionnaire. The ICD-10 codes used in this study are listed in Table 2.

**Table 2:** ICD-10 codes used for incident cancer cases

ICD-10 diagnose code	Name
DC18	Malignant neoplasm of colon
DC180 − DC189, excl. DC189X	Malignant neoplasm in various parts of colon, excluding relapse
DC20 + DC209, excl. DC209X	Malignant neoplasm of rectum, excluding relapse

### Statistical analyses

PPVs were calculated by dividing the number of symptomatic individuals diagnosed with CRC by the total number of symptomatic individuals in each category. NPVs were calculated by dividing the number of asymptomatic respondents not subsequently diagnosed with CRC by the total number of asymptomatic respondents. The PVs are presented as percentages. PPVs and NPVs for CRC were calculated for each category. Moreover, we calculated the positive likelihood ratios (LR+) and negative likelihood ratios (LR−) as a relative measure of the association between symptom experience and CRC. The PVs and LRs for CRC were calculated for each of the four specific alarm symptoms of CRC and for each of the six non-specific alarm symptoms for the total population (aged ≥40 years) and separately for two age groups 40–74 and≥75 years. For change in bowel patterns (stool texture and frequency), only experience of symptoms for the first time more than 1 month earlier was included. Moreover, the PVs and LRs were calculated for: (1) at least one of the ten alarm symptoms, (2) at least one of the specific alarm symptoms, (3) at least one of the non-specific alarm symptoms, (4) GP contact with at least one of the ten alarm symptoms and (5) GP contact with at least one specific alarm symptom. The PVs and LRs in these five groups were calculated for both the entire study population of respondents aged 40 years or above as well as for subgroups of individuals under and above 75 years of age. The proportions of specific and non-specific alarm symptoms of CRC reported by individuals aged 40 years or above diagnosed with CRC are presented.

All statistical tests used a significance level of *P* < 0.05. Data analyses were conducted using STATA statistical software 13.1 (StataCorp, College Station, TX, USA). In concordance with the Danish data protection act only summary tables with four or more individuals in each count are reported.

## Results

**Fig. 1:**
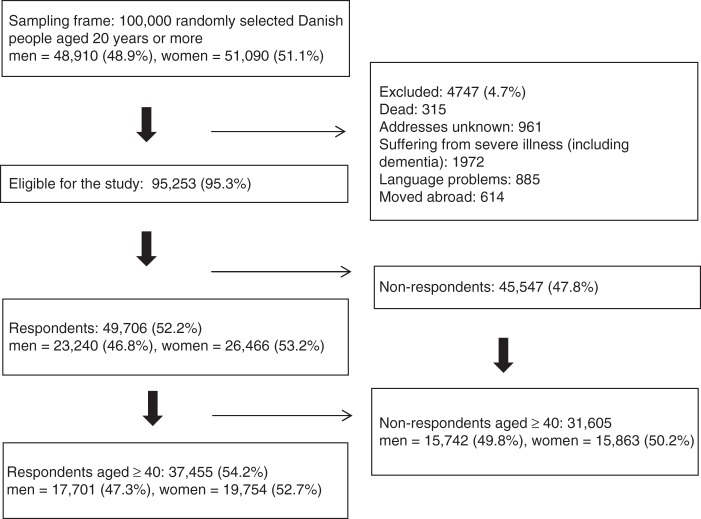
Study cohort

Of the 100,000 randomly selected subjects, 4474 (4.7%) were not eligible because they had either died, could not be reached due to unknown address, were suffering from severe illnesses, had language problems or had moved abroad. A total of 95,253 subjects were eligible for the study, of these 69,060 were aged 40 years or above and 37,455 completed the questionnaire, yielding an overall response rate of 54.2% (Fig. 1). The mean age of the respondents in this age group was 58.4 years (95% confidence interval (CI): 58.3–58.6) compared to 60.8 years (95% CI: 60.6–61.0) for non-respondents. Slightly more respondents were women (52.7%) compared to non-respondents (50.2%). The respondents were more often women married/co-habiting and had a higher income and educational level and were more often affiliated to the labour market (Table 5).

In total, 64 individuals (0.17%) were diagnosed with CRC 1 year after completing the questionnaire. Of these, 41 (64.1%) were males, and 23 (35.9%) were women. The mean age of the individuals diagnosed with CRC was 69.7 years (95% CI: 67.3–72.1).

Due to too few observations, only PVs for nine of the ten predefined symptoms are reported (i.e. PVs for unintended weight loss is not reported). The PPVs of the four specific alarm symptoms for CRC (abdominal pain, change in stool texture, change in stool frequency, blood in stool/rectal bleeding) ranged between 0.2% (95% CI: 0.1–0.4) (change in stool texture) and 0.6% (95% CI: 0.3–1.1) (rectal bleeding) (Table 3). The PPVs of the five non-specific alarm symptoms for CRC (diarrhoea, constipation, abdominal bloating, feeling unwell, tiredness) ranged between 0.1% (95% CI: 0.1–0.3) (constipation and feeling unwell) and 0.3% (95% CI: 0.1–0.5) (diarrhoea) (Table 3). The NPVs were 99.8 for all the specific and non-specific alarm symptoms (Table 3).

**Table 3:** Number of respondents (*n*, %), cases of colorectal cancers, numbers (*n*, %) (total numbers *n* = 64), PPVs (%) with 95% CI and LR+ and LR− with regard to symptom type (*n* = 37,455)

	Respondents, *n* (%)	Cancer cases, *n* (%)	PPV (95% CI)	LR+ (95% CI)	NPV (95% CI)	LR− (95% CI)
Specific alarm symptoms						
Abdominal pain	6223 (16.6)	17 (26.6%)	0.3 (0.2;0.4)	1.6 (1.1;2.4)	99.8 (99.8;99.9)	0.9 (0.8;1.0)
Change in stool frequency^a^	4207 (11.2)	10 (15.6%)	0.4 (0.2;0.7)	2.1 (1.2;3.7)	99.8 (99.8;99.9)	0.9 (0.8;1.0)
Change in stool texture^a^	5407 (14.4)	6 (9.4%)	0.2 (0.1;0.4)	1.0 (0.5;2.2)	99.8 (99.8;99.9)	1.0 (0.9;1.1)
Blood in stool/rectal bleeding	1378 (3.7)	8 (12.5%)	0.6 (0.3;1.1)	3.4 (1.8;6.5)	99.8 (99.8;99.9)	0.9 (0.8;1.0)
Non-specific alarm symptoms^b^						
Diarrhoea	4023 (10.7)	11 (17.2%)	0.3 (0.1;0.5)	1.6 (0.1;0.5)	99.8 (99.8;99.9)	0.9 (0.8;1.0)
Constipation	4945 (13.2)	7 (10.9%)	0.1 (0.1;0.3)	0.8 (0.4;1.7)	99.8 (99.8;99.9)	1.0 (0.9;1.1)
Abdominal bloating	9775 (26.1)	20 (31.3%)	0.2 (0.1;0.3)	1.2 (0.8;1.7)	99.8 (99.8;99.9)	0.9 (0.8;1.1)
Feeling unwell	4301 (11.5)	6 (9.4%)	0.1 (0.1;0.3)	0.8 (0.4;1.7)	99.8 (99.8;99.9)	1.0 (0.9;1.1)
Tiredness	16,282 (43.5)	26 (40.6%)	0.2 (0.1;0.2)	0.9 (0.7;1.3)	99.8 (99.8;99.9)	1.1 (0.9;1.3)

*PPV* positive predictive values, *LR+* positive likelihood ratios, *LR−* negative likelihood ratios, *CI* confidence interval

^a^Only experience of symptoms more than one month earlier was included

^b^Weight loss is missing because of too few observations

The PVs and LRs for CRC among individuals under and above 75 years are given in Table 4 and demonstrate the difference between age groups. Generally, the PPVs were lower and the NPVs higher in the group younger than 75 years compared to those aged 75 years and above. For the individuals aged 75 years or above who had reported at least one specific alarm symptom, a PPV of 1.0% (95% CI: 0.4–2.0) and an NPV of 99.5% (95% CI: 99.1–99.7) for CRC was estimated. Individuals aged 75 years or above reporting at least one of the specific alarm symptoms that lead to a GP contact had a PPV of 1.9% and LR+ of 3.0 for being diagnosed with CRC and the NPVs and LR− for this group was 99.5% (95% CI: 99.2–99.7) and 0.8% (95% CI: 0.6–1.0) respectively (Table 4).

**Table 4:** Number of respondents (*n*,%), cases of colorectal cancers, numbers (*n*, %), PPVs (%) and NPVs (%) with 95% CI and LR+  and LR− with 95% CI with regard to specific and non-specific cancer alarm symptoms and stratified on age group (under and above 75 years of age)

	Respondents, *n* (%)	Cancer cases, *n* (%)				
Total	37,455 (100%)	64 (100%)				
<75 years	34,323 (91.6%)	44 (68.8%)				
≥75 years	3132 (8.4%)	20 (31.2%)				
	Respondents, *n* (%)	Cancer cases, *n* (%)	PPV (95% CI)	LR+ (95% CI)	NPV (95% CI)	LR− (95% CI)
At least one alarm symptom						
Total	23,499 (62.7%)	42 (65.6%)	0.2 (0.1;0.2)	1.0 (0.9;1.2)	99.8 (99.8;99.9)	0.9 (0.7;1.3)
<75 years	21,766 (92.6%)	30 (71.4%)	0.1 (0.1;0.2)	1.1 (0.9;1.3)	99.9 (99.8;99.9)	0.9 (0.6;1.3)
≥75 years	1733 (7.4%)	12 (28.6%)	0.7 (0.4;1.2)	1.1 (0.8;1.6)	99.4 (98.9;99.8)	0.9 (0.5;1.5)
At least one specific alarm symptom						
Total	9925 (26.5%)	28 (43.8%)	0.3 (0.2;0.4)	1.7 (1.3;2.2)	99.9 (99.8;99.9)	0.8 (0.6;0.9)
<75 years	9206 (92.8%)	21 (75.0%)	0.2 (0.1;0.3)	1.8 (1.3;2.4)	99.9 (99.9;99.9)	0.7 (0.5;0.9)
≥75 years	719 (7.2%)	7 (25.0%)	1.0 (0.4;2.0)	1.5 (0.8;2.8)	99.5 (99.1;99.7)	0.8 (0.6;1.2)
At least one non-specific alarm symptom						
Total	22,343 (62.3%)	40 (62.5%)	0.2 (0.1;0.2)	1.0 (0.9;1.3)	99.8 (99.8;99.9)	0.9 (0.7;1.3)
<75 years	20,711 (92.7%)	29 (72.5%)	0.1 (0.1;0.2)	1.1 (0.9;1.4)	99.9 (99.8;99.9)	0.9 (0.6;1.3)
≥75 years	1632 (7.3%)	11 (27.5%)	0.7 (0.3;1.2)	1.1 (0.7;1.6)	99.4 (98.9;99.7)	0.9 (0.6;1.5)
Symptom experience and GP contact with at least one alarm symptom						
Total	6523 (17.4%)	20 (31.3%)	0.3 (0.2;0.5)	1.8 (1.2;2.6)	99.9 (99.8;99.9)	0.8 (0.7;1.0)
<75 years	5770 (88.5%)	13 (65.0%)	0.2 (0.1;0.4)	1.8 (1.1;2.8)	99.9 (99.8;99.9)	0.8 (0.7;1.0)
≥75 years	753 (11.5%)	7 (35.0%)	0.9 (0.4;1.9)	1.5 (0.8;2.7)	99.5 (99.1;99.7)	0.9 (0.6;1.2)
Symptom experience and GP contact with at least one specific alarm symptom						
<Total	2910 (7.8%)	14 (21.9%)	0.5 (0.3;0.8)	2.8 (1.8;4.5)	99.9 (99.8;99.9)	0.8 (0.7;1.0)
<75 years	2596 (89.2%)	8 (57.1%)	0.3 (0.1;0.6)	2.4 (1.3;4.5)	99.9 (99.8;99.9)	0.9 (0.8;1.0)
≥75 years	314 (10.8%)	6 (42.9%)	1.9 (0.7;4.1)	3.0 (1.5;6.0)	99.5 (99.2;99.7)	0.8 (0.6;1.0)

*PPV* positive predictive values, *NPV* negative predictive values, *LR+* positive likelihood ratios, *LR−* negative likelihood ratios, *CI* confidence interval

Of the individuals diagnosed with CRC, 43.8% had experienced at least one of the four specific alarm symptoms. Abdominal pain was the most common specific alarm symptom reported by 26.6% of the 64 individuals diagnosed with CRC. In total, 40.6% had reported tiredness, this being the most common non-specific symptom. Of the individuals diagnosed with CRC, 65.6 % had experienced at least one of the specific and non-specific alarm symptoms.

## Discussion

### Main findings

In this study, we investigated the PPVs, NPVs, LR+ and LR− of specific and non-specific alarm symptoms for CRC in the general population. The overall findings are that although the PPVs are low, the LR+s show a relative association with symptoms (e.g. LR+ 3.4 for rectal bleeding), and although the NPVs were high, the LR− estimates were too uncertain to conclude that individuals without symptoms are in no risk of CRC.

In general, the PPVs were higher among individuals aged 75 years and above compared to those aged 40–74 years. The NPVs were highest in the youngest age groups. The highest PPV was found among individuals aged 75 years and above reporting at least one of the specific alarm symptoms that led to a GP contact. The LR+ was substantially higher for individuals experiencing the specific alarm symptoms (LR+ 3.4% for rectal bleeding) when compared to the non-specific alarm symptoms (LR+ 1.2% for abdominal bloating). The lowest LR− was found for individuals aged 40–74 years reporting at least one specific alarm symptom.

### Strengths and limitations

The major strength of this study is the prospective cohort design following a large cohort of the general population, which gives the opportunity to retrieve information about symptom experiences prior to diagnosis. With this study design we minimised the risk of recall bias that is often seen in studies regarding cancer patients’ symptoms prior to diagnosis. The use of register-based diagnoses rather than asking the respondents further reduced the risk of recall bias. The DCR was used to identify cases of cancer. This registry is based on mandatory data from several sources and is a valid source of information on diagnoses.^18^

A general weakness of questionnaire-based studies is that respondents may not interpret the questions and categories of answers as intended. Prior to the survey, we conducted several rounds of pilot testing and field testing to reduce this possibility.^12^ Based on the results of the pilot testing, it seems reasonable to assume that the respondents understood the questions as intended.

This study reflects self-reported experience of symptoms within the preceding 4 weeks and subsequent contacts with a GP. Although we asked for symptom experiences and GP contacts within a short time period, some memory decay cannot be ruled out. Another limitation to keep in mind is the fact that willingness to respond to a questionnaire regarding symptom experiences may be associated with the presence of symptoms. It has been demonstrated that individuals with many or severe symptoms and vulnerable personality may be more prone to participate in a survey about symptoms.^19,20^

The age distribution of the cohort is skewed in the older age groups with regard to respondents and non-respondents (Table 5). However, as we do not know the symptom experience of the non-respondents we cannot know how this difference affects our results.

**Table 5:** Socio-demographic characteristics for the cohort of 69,060 individuals ≥40 years

	Total, *N* (%)	Respondents, *n* (%)	Non-respondents, *n* (%)
Total	69,060 (100.0%)	37,455 (100.0%)	31,605 (100.0%)
Sex			
Male	33,443 (48.4%)	17,701 (47.3%)	15,742 (49.8%)
Female	35,617 (51.6%)	19,754 (52.7%)	15,863 (50.2%)
Age groups			
40–59	36,187 (52.4%)	20,305 (54.2%)	15,882 (50.3%)
60–79	27,745 (40.2%)	15,748 (42.0%)	11,997 (38.0%)
80+	5128 (7.4%)	1402 (3.7%)	3726 (11.8%)
Marital status			
Single	21,191 (30.7%)	8423 (22.5%)	12,768 (40.5%)
Married/cohabiting	47,801 (69.3%)	29,008 (77.5%)	18,793 (59.5%)
Educational level			
Low (<10 years)	19,792 (29.5%)	8002 (21.7%)	11,790 (38.9%)
Middle (10–12 years)	28,959 (43.1%)	16,557 (45.0%)	12,402 (40.9%)
High (>12 years)	18,386 (27.4%)	12,263 (33.3%)	6123 (20.2%)
Labour market affiliation			
Working	37,603 (54.5%)	22,930 (61.2%)	14,673 (46.5%)
Retirement pension	23,193 (33.6%)	11,294 (30.2%)	11,899 (37.7%)
Out of workforce	8228 (11.9%)	3222 (8.6%)	5006 (15.9%)
Equivalence weighted disposable income			
Low (1st quartile)	13,598 (19.7%)	4498 (12.0%)	9100 (28.8%)
Middle (2nd and 3rd quartile)	35,122 (50.9%)	19,309 (51.6%)	15,813 (50.1%)
High (4th quartile)	20,272 (29.4%)	13,624 (36.4%)	6648 (21.1%)
Ethnicity			
Danish	64,418 (93.4%)	35,609 (95.1%)	28,809 (91.3%)
Immigrants and descendants of immigrants	4574 (6.6%)	1822 (4.9%)	2752 (8.7%)

Missing values in each category do not exceed 10%

We investigated whether non-respondents had a higher number of CRC and found slightly more cases of CRC among non-respondents in the age group ≥40 years (64 cases among the 37,455 (0.17%) respondents compared to 72 cases among the 31,605 non-respondents (0.23%)). The differences in the number of CRCs in the two groups might be affected by a number of factors. Firstly, the non-responders were slightly older than the respondents. Since the incidence of CRC increases with age, this might be an associated factor. Older age might be associated with a higher degree of comorbidity and therefore lower capacity to participate in the survey. Secondly, it could be due to socioeconomic factors. A Danish study from 2008 found that higher incidences of CRC were associated with social disadvantages predominantly amongst men, specifically related to co-habiting status, housing tenure, dwelling size and affiliation to the labour market.^21^ The non-respondents in our study had lower socioeconomic status compared to respondents. A third possible associated factor could be that people who take their symptoms less seriously are more likely to have CRC.^22^ As the non-respondents did not wish to participate in the survey on symptoms and healthcare-seeking, one could hypothesise that they were less likely to take symptoms seriously.

The limited number of cases of CRC might affect the reliability of the results. Due to the limited number of cases of CRC in the follow-up period, we did not have sufficient power to evaluate the PVs of combinations of alarm symptoms. It is plausible that experiencing more than one alarm symptom increases the risk of malignancy.^23^ From previous studies we know that experiencing multiple symptoms increases the probability of seeking medical attention.^15^ Therefore, the non-specific symptoms of CRC might play an important role in the diagnostic pathway.

### Comparison with previous literature

To our knowledge, this is the only population-based study estimating PPVs and NPVs of alarm symptoms of CRC with corresponding LR+ and LR−. A literature study by Fijten et al.,^24^ which includes nine population-based studies, estimated the PPV of rectal bleeding to be 1 in 1000. A relatively new systematic review investigating the diagnostic value of rectal bleeding found PPVs ranging from 0.01 to 0.21%.^25^ However, this review was comprised of studies from various settings and all the population-based studies targeted screening of CRC.

Several studies have investigated the diagnostic values of alarm symptoms of CRC in primary care^8,23,26^ and found PVs highest for rectal bleeding and anaemia. This is in line with the present results where the single symptom with highest PPV was rectal bleeding. The PPVs in the studies from general practice were substantially higher. A review by Astin et al.^23^ found that PPVs for rectal bleeding as single symptom varied between 2.2 and 15.8%. The findings in the present study support this suggestion, as alarm symptoms presented to the GP had a higher PPV compared to alarm symptoms not reported to the GP. This indicates that deciding to consult the GP with a symptom increases the likelihood of the symptom being caused by a disease.

In a previous study, we have demonstrated that no more than 33.8% of the individuals in the general population who experienced rectal bleeding consulted their GP.^7^ This in line with the findings from Crosland and Jones^27^ who found that 41% of people from the general population had consulted their GP when experiencing rectal bleeding. These results combined with results from the present study have clear implications for modelling the impact and cost-effectiveness for public awareness campaigns. Unfortunately, awareness campaigns have not yet shown to improve survival.^28^ Although screening has been implemented in some countries, most CRC patients are still to be found based on symptoms. Instead of general awareness campaigns, targeted education of older persons, who have the highest incidence, might have an effect on healthcare-seeking and ultimately survival of CRC. It is important that we communicate the importance of healthcare-seeking with the defined alarm symptoms and the fact that low risk is not no risk. Informing patients referred for fast track investigation of CRC about the modest risk of actually having CRC could possibly reduce the anxiety related to investigation for suspected cancer.^29^

## Conclusions

This study supports our hypothesis that PPVs of alarm symptoms of CRC experienced in an unselected general population were lower than PPVs of symptoms presented in hospitals or general practices. The single symptom with highest PPV was rectal bleeding. The fact that rectal bleeding as an initial symptom is associated with a less advanced stage of CRC and increased survival rate^9^ supports the idea that future campaigns aiming to promote early diagnosis should focus on increasing healthcare-seeking when experiencing rectal bleeding. Individuals aged 75 years and above who reported having contacted the GP with the alarm symptoms had the highest PPV. This finding gives reason to further explore the process of people’s decision to seek healthcare and to target education of older persons, who have the highest incidence of CRC.

The PPVs for the alarm symptoms were low and the NPVs were high. Experiences of specific and non-specific alarm symptoms are frequent. This means that despite requiring further investigation, most patients with an alarm symptom of CRC do not have CRC.

## Supplementary information

Legend Supplementary Figure 1

Supplementary Figure 1

## Data Availability

The datasets generated and analysed during the current study are not publicly available due to the data protection regulations of the Danish Data Protection, Statistics Denmark and the Danish Health and Medicines Authority. Access to data is strictly limited to the researchers who have obtained permission for data processing. This permission was granted to the Research Unit of General Practice, Department of Public Health, University of Southern Denmark.
