# Supplementary material for: Predictive values of colorectal cancer alarm symptoms in the general population: a nationwide cohort study
Source: Br J Cancer. 2019 Feb 22;120(6):595–600. doi: 10.1038/s41416-019-0385-x (PMC6461905; doi:10.1038/s41416-019-0385-x)
Supplement: Supplementary file 1 — Legend Supplementary Figure 1 [file 41416_2019_385_MOESM1_ESM.docx]

Supplementary Figure 1: Guidelines for referral of patients suspected of colorectal cancer
