# Supplementary material for: Predictive values of colorectal cancer alarm symptoms in the general population: a nationwide cohort study
Source: Br J Cancer. 2019 Feb 22;120(6):595–600. doi: 10.1038/s41416-019-0385-x (PMC6461905; doi:10.1038/s41416-019-0385-x)
Supplement: Supplementary file 2 — Supplementary Figure 1 [file 41416_2019_385_MOESM2_ESM.docx]

| There is a reasonable suspicion of cancer of the colon and rectum in all patients over 40 years with one or several of the following symptoms:  • Bleeding from the intestine /rectal bleeding  • Change in an otherwise stable stool pattern for more than four weeks  • Unexplained bleeding anaemia  • Significant general symptoms (e.g. abdominal pain) |
| --- |
